# Acral melanoma: Molecular subtyping as a gateway to precision medicine

**DOI:** 10.1111/jdv.20736

**Published:** 2025-05-20

**Authors:** Emi Dika, Elisabetta Magnaterra

**Affiliations:** ^1^ Department of Medical and Surgical Sciences (DIMEC) University of Bologna Bologna Italy; ^2^ Oncologic Dermatology Unit IRCCS Azienda Ospedaliero‐Universitaria di Bologna Bologna Italy

Acral melanoma (AM) is a uniquely challenging subtype of melanoma, often diagnosed at advanced stages and with limited therapeutic options.^1^, ^2^ Its distinct biological and clinical behaviour, as well as its rarity in Caucasian populations, have long hindered large‐scale molecular studies. Prior limited single‐center studies have shown variable somatic mutational patterns of AM and have not concluded towards any correlation with prognosis or clinical behaviour.^3^ This inconsistency has underscored the need for alternative strategies to characterize AM at the molecular level.

The recent work by Yin et al.^4^ marks a significant advance in our understanding of AM by identifying two molecularly and prognostically distinct subtypes using an integrative multi‐omics approach.

Using an integrative analysis of bulk and single‐cell RNA sequencing data, enhanced by a network‐based gene prioritization approach (CIPHER/CIPHER‐SC), the authors classified AM into two biologically and clinically distinct subtypes. Subtype I was associated with thinner tumours, a more immune‐inflamed microenvironment and better prognosis, while Subtype II showed macrophage‐driven immunosuppression and worse outcomes. To support this classification, they identified a minimal gene panel composed of EREG, predominantly expressed in Subtype I and RAB20, FCGR3A and VSIG4, specifically upregulated in Subtype II. This four‐gene signature demonstrated strong discriminatory power and may serve as a useful tool for patient stratification in future research and clinical applications.

Beyond its technical innovation, this study underscores a broader paradigm shift. Transcriptomic technologies—particularly at the single‐cell level—are redefining our understanding of tumour biology, especially in underexplored entities like AM. As our knowledge of AM pathogenesis expands, so too does the opportunity to develop targeted therapies and predictive biomarkers of immunotherapy response. The distinct immune landscapes described—especially the divergent roles of FCN1^+^ and SPP1^+^ macrophages—open new avenues for therapeutic intervention, including macrophage modulation strategies.^5^

Nonetheless, several questions remain open. The clinical relevance of AM warrants further exploration. Are there associations between subtypes and anatomical localization—for instance, a predilection of Subtype II for the nail apparatus melanoma, which has been associated with more aggressive behaviour in previous studies? Does histopathological subtype (acral lentiginous vs. nodular or superficial spreading) correlate with molecular classification? The authors do not stratify their analysis by histopathologic subtype, which could add important context given the known heterogeneity of AM even within acral sites.

Furthermore, while the use of high‐throughput sequencing and computational methods is laudable, the costs and complexity of such technologies remain non‐trivial. Widespread adoption in clinical practice will require validation in larger, prospective cohorts and the development of streamlined assays, ideally applicable to formalin‐fixed tissue. It is encouraging, however, that the biomarker panel described consists of only four genes, which may be adaptable to immunohistochemistry or RT‐qPCR platforms in the near future.

Ultimately, whether and how this molecular framework will translate into improved patient care remains to be seen. Still, it offers a promising foundation for future research and a step forward in addressing the biological complexity of a melanoma subtype that has long remained underexplored.

## CONFLICT OF INTEREST STATEMENT

E.D. is on the advisory board or has received honoraria from Regeneron, Novartis, Difacooper, Bristol‐Myers Squibb, MSD Sharp &Dohme, Pierre‐Fabre Pharma, Sanofi Genzyme, SUN Pharma and travel support from SUN Pharma, outside the submitted work. E.M. has nothing to disclose.

## Data Availability

Data sharing is not applicable to this article as no new data were created or analysed in this study.

## References

[1] Elder DE , Bastian BC , Cree IA , Massi D , Scolyer RA . The 2018 World Health Organization classification of cutaneous, mucosal, and uveal melanoma: detailed analysis of 9 distinct subtypes defined by their evolutionary pathway. Arch Pathol Lab Med. 2020;144(4):500–522. 10.5858/arpa.2019-0561-RA 32057276

[2] Durbec F , Martin L , Derancourt C , Grange F . Melanoma of the hand and foot: epidemiological, prognostic and genetic features. A systematic review. Br J Dermatol. 2012;166(4):727–739. 10.1111/j.1365-2133.2011.10772.x 22175696

[3] Dika E , Veronesi G , Altimari A , Riefolo M , Ravaioli GM , Piraccini BM , et al. BRAF, KIT, and NRAS mutations of Acral melanoma in white patients. Am J Clin Pathol. 2020;153(5):664–671. 10.1093/ajcp/aqz209 32017841

[4] Yin M , Zhang Y , Wang W , Zhao S , Su J , Li S , et al. Identification of two molecularly and prognostically distinct subtypes in acral melanoma using network prediction method. J Eur Acad Dermatol Venereol. 2025;39:1254–1266. 10.1111/jdv.20335 PMC12188512 39282977

[5] Guerriero JL , Sotayo A , Ponichtera HE , Castrillon JA , Pourzia AL , Schad S , et al. Class IIa HDAC inhibition reduces breast tumours and metastases through anti‐tumour macrophages. Nature. 2017;543(7645):428–432. 10.1038/nature21409 28273064 PMC8170529

